# Knowledge, attitude and practice on dengue prevention and dengue seroprevalence in a dengue hotspot in Malaysia: A cross-sectional study

**DOI:** 10.1038/s41598-020-66212-5

**Published:** 2020-06-12

**Authors:** Sivaneswari Selvarajoo, Jonathan Wee Kent Liew, Wing Tan, Xin Ying Lim, Wardha F. Refai, Rafdzah Ahmad Zaki, Neha Sethi, Wan Yusoff Wan Sulaiman, Yvonne Ai Lian Lim, Jamuna Vadivelu, Indra Vythilingam

**Affiliations:** 1 Department of Parasitology, Faculty of Medicine, University of Malaya, 50603 Kuala Lumpur, Malaysia; 2 Postgraduate Institute of Medicine (PGIM), Colombo, 7 Sri Lanka; 3 Centre for Epidemiology and Evidence Based Practice, Department of Social and Preventive Medicine, Faculty of Medicine, University of Malaya, 50603 Kuala Lumpur, Malaysia; 4 Department of Obstetrics and Gynaecology, University of Malaya Medical Centre (UMMC), 50603 Kuala Lumpur, Malaysia; 5 Department of Medical Microbiology, Faculty of Medicine, University of Malaya, 50603 Kuala Lumpur, Malaysia

**Keywords:** Entomology, Viral infection

## Abstract

Dengue has become a global public health problem. Despite reactive efforts by the government in Malaysia, the dengue cases are on the increase. Adequate knowledge, positive attitude and correct practice for dengue control are essential to stamp out the disease. Hence, this study aims to assess the factors associated with dengue knowledge, attitude and practice (KAP), as well as the association with dengue IgM and IgG seropositivity. A community-based cross-sectional study was conducted in a closed, dengue endemic area with multi-storey dwellings . Five hundred individuals (aged 18 years and above) were approached for pre-tested KAP and seroprevalences assessment. The study showed only half of the total participants have good knowledge (50.7%) but they had insufficient knowledge about dengue during pregnancy. 53.2% of people had poor attitude and 50.2% reported poor practice for dengue control. Out of 85 respondents who agreed to participate in the dengue seroprevalence study, 74.1% (n = 63) were positive for dengue IgG and 7.1% (n = 6) were positive for dengue IgM. Among all sociodemographic variable, race is the only independent predicator for all KAP levels (P < 0.05). In conclusion, proactive and sustainable efforts are needed to bring a behavioural change among communities in order to fight dengue outbreaks in endemic areas.

## Introduction

Dengue fever is a mosquito-borne viral disease caused by a flavivirus. There are four distinct serotypes of dengue virus, namely DEN-1, 2, 3 and 4. Female *Aedes aegypti* and *Aedes albopictus* mosquitoes are the primary and secondary vectors in Malaysia, respectively. Evidently, dengue is the most rapidly spreading arboviral disease in the world. The Global Burden of Disease reported that dengue incidence has multiplied to six-folds from 1990 to 2013, with Southeast Asia region contributing 52% of the disease burden^1^. World Health Organisation (WHO) estimates that 50 million to 100 million cases occur annually^2^.The disease is currently endemic in more than 100 countries, with South-East Asia being among the worst affected region.

Dengue fever was established in Malaysia ever since the first reported case of dengue in 1902. From then on, the numbers of cases continued to rise despite numerous initiatives undertaken by the Ministry of Health to curb the disease^3,4^. According to WHO, the recent cumulative case count in Malaysia from 1 Jan to 2 Mac 2019 was 157% higher than that of the same period in 2018^5^. In addition, a total of 79,151 dengue cases have been reported until end of July 2019 nationwide, with Selangor state contributing more than 50% of the cases (n = 40,849, 51.6%)^6^.

Vector control and surveillance is still the mainstay of dengue prevention strategies since there is no specific treatment for disease and vaccination remains a non-viable option^7^. Local programs like Communications for Behavorial Changes (COMBI) in Malaysia have proved their potential effect in reducing dengue morbidity^8^ but it requires understanding from community as well^9^. Besides, vector control measures eg. larval survey, fogging, ULV sprays and laws such as the Destruction of Disease Bearing Insects (Amendment) Act 2000, require support, cooperation and participation from the community^10^. Therefore, an understanding of the society’s baseline knowledge, attitudes and practices (KAP) of dengue is essential for effective vector control. Health education is equally important in the prevention of dengue^11–13^. Hence, apart from evaluating the KAP of the community with regards to dengue, providing basic knowledge of the disease and its preventive methods is of paramount importance.

In 1997, two cases of vertical transmission of dengue fever in Malaysia were reported for the first time^14^. A total of 16 dengue cases in pregnancy were reported in a study conducted in Malaysia from 2000 to 2004, which concluded that dengue infection in pregnancy may lead to poor maternal and foetal outcomes^15–17^. A hospital-based prospective study conducted in Vientiane, Laos found dengue to be the most common infection among febrile pregnant women^18^. Symptomatic dengue infection during pregnancy or delivery may even lead to preterm births, infants with low birth weight^19^, haemorrhagic complications, maternal death, vertical transmission of dengue to symptomatic infants, and other neonatal complications^20^. Since prompt treatment and admission are needed, the community should be more aware of the impact of dengue during pregnancy. However, to date no data has been reported on the community’s knowledge regarding dengue in pregnancy. Therefore, this study also provides an opportunity to assess and clarify any misconceptions regarding dengue infection in pregnancy among the community.

Of the 390 million DENV infections per year, 300 million are asymptomatic^21^. As primary infections by the virus are usually asymptomatic, the actual magnitude of dengue infection in Malaysia is likely to be greater than expected. Interestingly, a nationwide cohort study found that 91.6% of the adults aged 35 to 74 years were dengue IgG positive^22^. Considering, the entire discourse above, this study also aimed to investigate the factors which affect the dengue related KAP of an endemic community, as well as the association of the KAP with dengue IgM and IgG seropositivity.

Various studies which have been conducted in different parts of Malaysia primarily focused on sociodemographic data and KAP of study populations^23^. Previous studies performed in Selangor, where the current study took place, demonstrated that the general communities have good knowledge on dengue and positive attitudes when it comes to dengue prevention^23–26^. These factors are further differentially influenced by age group, ethnicity, level of education, employment status and marital status^27,28^. KAP aside, several other researches have also evaluated the association between the health belief model construct and IgG seropositivity in Malaysians^29^. To the best of our knowledge, there are hardly any studies on KAP and associated factors of dengue infections in closed, endemic communities. Also, in view of the recent rise in dengue deaths within the study area, it is a necessity to assess the dengue related KAP of the endemic community to devise effective vector control and surveillance strategies.

## Results

### Sociodemographic characteristics of study population

Out of 500 questionnaires distributed among residents of eight apartments, 474 responded, giving a response rate of 94.8%. Table 1 shows the demographic status of the participants. Briefly, the study population is predominated by females (60.6%) with mean age of 36 ± 11.62. Majority of the respondents were Malay (62.1%) and married (74.4%). More than half of the participants (56.6%) were residing in low rise apartments. Almost all participants had received primary education (91.8%). Of the total respondents, 51.8% of them were working and earning an individual monthly income of less than Malaysian Ringgit (MYR) 5,000 (94.1%) (1 USD = MYR 4.15).

**Table 1:** Sociodemographic characteristics of study population.

Characteristic		n (%)
Gender (n = 421)	Male	166 (39.4)
	Female	255 (60.6)
Age group (n = 395)	≤35	206 (52.2)
	>35	189 (47.8)
Race (n = 419)	Malay	260 (62.1)
	Chinese	43 (10.3)
	Indian	90 (21.5)
	Others	26 (6.2)
Marital status (n = 418)	Single	96 (23.0)
	Married	311 (74.4)
	Widowed	11 (2.7)
Education (n = 407)	None	5 (1.2)
	Primary	29 (7.0)
	Secondary	218 (52.7)
	Tertiary	162 (39.1)
Occupation (n = 408)	Working	236 (51.8)
	Not working	171 (41.9)
Income (n = 390)	<MYR 1,000	193 (48.1)
	MYR 1,001–3,000	127 (31.7)
	>MYR 3,001	81 (20.1)
Type of residential buildings (n = 419)	High rise^a^	97 (23.2)
	Low rise^b^	237 (56.6)
	Shop house^c^	85 (20.3)
History of dengue (n = 413)	Yes	69 (16.7)
	No	344 (83.3)
Dengue IgG level (n = 85)	Positive	63 (74.1)
	Negative	22 (25.9)
Dengue IgM level (n = 85)	Positive	6 (7.1)
	Negative	79 (92.9)

^a^High rise is defined as buildings with more than five floors which require the use of elevator. ^b^Low rise is defined as buildings with up to five floors with absence of an elevator. ^c^Shop house is defined as residential units above the shop lots. MYR, Malaysian Ringgit; (USD 1 = MYR 4.15).

Despite being marked as a hotspot area for dengue, 83.3% respondents self-reported that they never had any history of dengue, only 16.7% had been infected with dengue in the past. Among 474 respondents, only 85 respondents agreed to participate in the dengue seroprevalence study. Based on this, it is a mere 18% of the total respondents, out of which 74.1% (n = 63) were positive for dengue IgG and 7.1% (n = 6) were positive for dengue IgM. Dengue IgM positivity demonstrates a recent or acute dengue infection, while dengue IgG positive individuals have had previous exposure to dengue. Therefore, in this community where mostly did not report any symptom at the time of sampling, 74.1% of the participants had previous exposure to dengue, while 7.1% had recent or acute dengue infection.

### Knowledge about dengue

Table 2 summarizes the correct knowledge of respondents on dengue. Among the endemic study population, level of knowledge regarding dengue disease and transmission is high. Almost all the questions in the “knowledge” category were answered correctly by more than half of the study population. Most respondents knew dengue to be a viral disease (82.2%) and is transmitted by mosquito bites (97.2%). Furthermore, majority of participants were able to respond correctly to questions on vector behaviour, regarding active biting time and breeding preferences. However, knowledge regarding dengue during pregnancy was lacking among the study participants. Only 54.6% were aware that dengue virus can be transmitted from an infected pregnant mother to the foetus. Moreover, knowledge regarding the life cycle of dengue vectors was also not satisfactory. Only 18% were able to answer correctly the number of days required by the vector mosquitoes to complete their life cycle. In addition, only two-thirds (66.7%) were aware that dengue virus was able to infect the same person multiple times. Regarding symptoms of dengue, high grade fever was a popular response among study subjects followed by muscle pain (92.6%), headache (92.5%) and joint pain (91.0%). However, rapid breathing (70.7%) and restlessness (71.1%) were less identified as associated symptoms of dengue fever. Overall, based on 80% cut-off value, 50.7% participants (n = 213) were identified to have substantial knowledge of dengue.

**Table 2:** Knowledge about dengue.

Statement		Correct knowledge, n (%)
Dengue is caused by a virus (n = 399)		328 (82.2)
Dengue is transmitted by mosquito bite (n = 423)		411 (97.2)
Dengue virus can be transmitted from infected pregnant mother to the baby. (n = 409)		260 (54.6)
Dengue infection increases risk of miscarriage (n = 409)		293(71.6)
Pregnant women with dengue infection have higher risk of complication compared to pregnant women without dengue (n = 409)		353 (86.3)
*Aedes aegypti* and *Aedes albopictus* are main vectors for dengue in Malaysia (n = 396)		357 (90.2)
*Aedes* mosquito prefers to breed in clean water (n = 415)		278 (58.4)
*Aedes* mosquito can breed both indoor and outdoor (n = 414)		371 (89.6)
Clean water as little as 5 mL is enough for mosquito to breed (n = 391)		345 (88.2)
Life cycle (Eggs-larvae-pupae-adult) (n = 401)		378 (94.3)
10 days are required for complete maturation of mosquito from eggs to adults (n = 384)		69 (18.0)
Eggs can survive in dry condition up to 6 months (n = 389)		204 (52.4)
All fish cannot be used to kill *Aedes* mosquito’s larvae (n = 392)		213 (54.4)
Mosquito likes to bite early in the morning and late evening (n = 403)		370 (91.8)
Dengue fever can cause death (n = 405)		377 (93.1)
You and your family members are at risk of dengue (n = 410)		265 (64.6)
The same person can be infected with dengue more than once (n = 408)		272 (66.7)
**Sign of dengue**	High grade fever (n = 408)	380 (93.1)
	Headache (n = 405)	375 (92.5)
	Muscle pain (n = 405)	375 (92.6)
	Joint pain (n-400)	365 (91.0)
	Rashes (n = 403)	345 (85.6)
	Pain behind the eyeball (n = 395)	301 (76.2)
	Persistent vomiting or diarrhoea (n = 404)	343 (84.9)
	Rapid breathing (n = 396)	280 (70.7)
	Severe abdominal pain (n = 391)	292 (74.7)
	Bleeding from nose or gums (n = 398)	294 (73.9)
	Restlessness (n = 392)	280 (71.1)

Factors associated with good knowledge (Table 3) were age, race, marital status, monthly income, education and employment (P < 0.05). Whereas, gender and previous history of dengue have no association with participant’s knowledge. Our findings revealed participants with higher income and education background have good knowledge compared to those with lower income and education. Moreover, participants more than 35 years and married have better knowledge about dengue. Interestingly, Malays are 2.3 times more likely to have good knowledge compared to non-Malays. After all significant factors (P ≤ 0.25) were included in analysis, multivariable model (Table 4) revealed race and occupation as the independent predictors for good knowledge.

**Table 3:** Univariate predictors of good KAP.

Characteristic		Knowledge		Attitude		Practice	
		cOR	P	cOR	P	cOR	P
Gender	Male	1.00					
	Female	1.15 (0.77–1.70)	0.495	1.12 (0.76–1.66)	0.570	1.16 (0.79–1.72)	0.448
Age group	≤35	1.00					
	>35	1.30 (0.87–1.94)	0.198	1.56 (1.05–2.32)	0.030*	0.98 (0.66–1.46)	0.939
Race	Malay	2.33 (1.55–3.51)	<0.001*	2.23 (1.48–3.36)	<0.001*	3.08 (2.03–4.67)	<0.001*
	Non- Malay	1.00					
Marital status	Single	1.00					
	Married	1.76 (1.12–2.77)	0.014*	2.44 (1.53–3.89)	<0.001*	1.46 (0.93–2.27)	0.097
Education level	No formal education or primary education	1.00					
	Secondary education and beyond	2.36 (1.08–5.15)	0.031*	2.08(0.96–4.50)	0.064	1.57 (0.75–3.26)	0.231
Occupation	Working	1.78 (1.19–2.64)	0.005*	1.68 (1.13–2.50)	0.010*	1.37 (0.92–2.02)	0.118
	Not working	1.00					
Income	≤RM 5,000	1.00					
	>RM 5,000	3.54 (1.29–9.74)	0.014*	3.24 (1.25–8.41)	0.016*	2.37 (0.95–5.90)	0.063
IgG level	Positive	1.00					
	Negative	1.17 (0.44–3.16)	0.752	1.41 (0.53–3.73)	0.492	1.33 (0.50–3.53)	0.562
IgM level	Positive	1.00					
	Negative	1.03 (0.20–5.41)	0.976	1.95 (0.34–1.26)	0.455	4.41 (0.49–3.94)	0.185

cOR, Crude odds ratio (at 95% confidence interval).

*Significant P value (P < 0.05).

**Table 4:** Multivariate analysis predictors of good KAP.

Characteristic		Knowledge		Attitude		Practice	
		aOR	P	aOR	P	aOR	P
Age group	≤35						
	>35	1.25 (0.79–2.00)	0.343	1.20 (0.82–2.07)	0.264		
Race	Malay	1.92 (1.22–3.01)	0.005*	1.98 (1.26–3.13)	0.003*	2.89 (1.86–4.49)	<0.001*
	Non- Malay						
Marital status	Single						
	Married	1.35 (0.79–2.33)	0.275	1.78 (1.03–3.10)	0.040*	1.404 (0.86–2.30)	0.178
Education level	No formal education or primary education						
	Secondary education and beyond	2.17 (0.88–5.35)	0.090	1.91 (0.78–4.70)	0.159	1.04 (0.46–2.34)	0.927
Occupation	Working	1.78 (1.15–2.79)	0.010*	1.70 (1.09–2.65)	0.020*	1.38 (0.90–2.12)	0.140
	Not working						
Income	≤RM 5,000						
	>RM 5,000	3.08 (0.99–9.59)	0.052	2.70 (0.94–7.78)	0.066	1.96 (0.75–5.07)	0.168

IgG and IgM seroprevalence were not included in the regression model due to small sample size. aOR, Adjusted odds ratio (at 95% confidence interval). *Significant P value (P < 0.05).

### Attitude toward dengue prevention

Table 5 shows the participants’ attitude towards dengue prevention. 89.7% of participants would like to reduce dengue cases in their area. However, not all of them (70.5%) regularly check the dengue situation in their areas. Around half of them have the wrong perception that chemical fogging by health authorities is enough for dengue prevention. Only 78.0% would like to actively engage in removal of breeding sites. Based on 80% cut off value, 46.8% of the study population possess an appropriate and acceptable attitude towards dengue prevention and this is associated with age, race, marital status and employment (Table 3). In multivariable model, employment, marital status and race were independent factors significantly associated with good attitude (Table 4).

**Table 5:** Attitude towards dengue prevention.

Variable		n (%)
I want to help to reduce the number of dengue cases in my area (n = 397)	Yes	365 (89.7)
	No	11 (2.8)
	Not sure	30 (7.6)
I check dengue situation or hotspots around my area regularly (n = 396)	Yes	279 (70.5)
	No	117 (29.5)
I will take extra action to prevent dengue infection if I know the risk of being infected with dengue is increasing in my area (n = 392)	Yes	346 (88.3)
	No	10 (2.6)
	Not sure	36 (9.2)
Removal of mosquito breeding sites at my premises will reduce the chance of dengue infection among my family members. (n = 394)	Yes	351 (89.1)
	No	15 (3.8)
	Not sure	28 (7.1)
Chemical fogging by health authority is good enough to prevent dengue infection (n = 397)	Yes	197 (49.6)
	No	128 (32.2)
	Not sure	72 (18.1)
It is not my responsibility to remove mosquito breeding sites in my residences (n = 394)	Yes	74 (18.8)
	No	320 (81.2)
It is necessary to continue the removal of mosquito breeding sites at home even during the period when there’s no outbreak (n = 392)	Yes	359 (91.6)
	No	13 (3.3)
	Not sure	20 (5.1)
Dengue outbreak in my community can be controlled if every household is committed to remove mosquito breeding sites (n = 391)	Yes	363 (92.9)
	No	9 (2.3)
	Not sure	19 (4.9)
I will take part in a public activity for dengue control or removal of mosquito breeding sites	Yes	305 (78.0)
	No	14 (3.6)
	Not sure	72 (18.4)

### Practices toward dengue prevention

Table 6 shows participants’ practices towards preventing dengue. In general, 50.2% of participants have unsatisfactory practice towards dengue prevention based on 80% cut-off. The response “search and destroy mosquito breeding sites” (95.0%) followed by “usage of mosquito spray” (94.5%) were much preferred methods of control by participants whenever there was a high abundance of mosquitoes. About 53.5% of them were aware of scrubbing containers before discarding the containers with water collections to get rid of mosquito eggs attached to containers. The best self-protection method was chosen as removal of mosquito breeding sites (80.4%) followed by 58.3% preferring to use mosquito repellents. Race is the only factor correlated with good prevention practice for dengue (Table 4). Malay participants were three times more likely to have good prevention practice for dengue compared to non-Malays.

**Table 6:** Practices of dengue prevention.

Variable		n (%)	
Calling health authority for fogging (n = 402)	Yes	372 (92.5)	
	No	30 (7.5)	
Calling private pest control (n = 392)	Yes	240 (61.2)	
	No	152 (38.8)	
Search and destroy breeding site (n = 401)	Yes	381 (95.0)	
	No	20 (5.0)	
Use mosquito spray (n = 396)	Yes	374 (94.5)	
	No	22 (5.6)	
Which best describes Search and Destroy? (n = 476)	Discard water with larvae	55 (14.1)	
	Discard water with larvae and wash container with antiseptic	96 (24.7)	
	Discard stagnant water and scrub the container	208 (53.5)	
	Discard stagnant water and wash container with hot water	30(7.7)	
Which method do you think is best to protect yourself and your family members from dengue infection (n = 398)	Mosquito repellent	Yes	232 (58.3)
		No	166 (41.7
	Bed nets	Yes	198 (49.7)
		No	200 (50.3)
	Remove breeding sites	Yes	320 (80.4)
		No	78 (19.6)
	Insecticide	Yes	155 (39.0)
		No	243 (61.1)
In your opinion, which is the most effective method to reduce dengue infection in your area?	Search & destroy mosquito breeding sites	332 (85.8)	
	Prevent from mosquito bites	14 (3.6)	
	Chemical fogging	37 (9.6)	

### Factors associated with dengue IgG seropositivity

Table 7 shows the sociodemographic factors associated with dengue IgG seropositivity. Type of residential units and ethnicity were significantly associated with IgG seropositivity. IgG seropositivity in 73.2% of 85 participants suggests they were unknowingly infected with dengue virus previously. Interestingly, 75.0% of the participants with positive dengue IgG possessed poor knowledge, attitude and practice toward dengue disease.

**Table 7:** Factors associated with IgG seropositivity.

Characteristic		Presence of IgG, n (%)	Absence of IgG, n (%)	P
Gender (n = 85)	Male	24 (72.7)	9 (27.3)	0.816
	Female	39 (75.0)	13 (25)	
Age group (n = 82)	≤35	33 (68.8)	15 (31.3)	0.204
	>35	28 (82.4)	6 (17.6)	
Race (n = 84)	Malay	33 (68.8)	15 (31.3)	0.037*^Φ^
	Chinese	8 (57.1)	6 (42.9)	
	Indian	15 (100)	0	
	Others	6 (85.7)	1 (14.3)	
Marital status (n = 84)	Single	18 (72)	7(28)	0.851^Φ^
	Married	43(75.4)	14(24.6)	
	Widowed	2 (100)	0	
Education level (n = 84)	No formal education or primary education	9 (100)	0	0.067
	Secondary education and beyond	54 (72)	21 (28)	
Occupation (n = 81)	Working	39 (76.5)	12 (23.5)	0.521
	Not working	21 (70)	9 (30)	
Income (n = 78)	≤MYR 5,000	52 (76.5)	16 (23.5)	0.078^Φ^
	>MYR 5,000	5 (50)	5 (50	
Type of Buildings (n = 85)	High rise	8 (47.1)	9(52.9)	0.017*
	Low rise	45(80.4)	11 (19.6)	
	Shop house	10 (83.3)	2 (9.1)	
Self-reported of dengue (n = 83)	Yes	10 (83.3)	2 (16.7)	0.457^Φ^
	No	52(73.2))	19 (26.8)	
Knowledge level (n = 83)	Good	30(73.2)	11(26.8)	0.804
	Poor	32(76.2)	10(23.8)	
Attitude level (n = 85)	Good	29 (70.7)	12 (29.3)	0.491
	Poor	34 (77.3)	10 (22.7)	
Practice level (n = 85)	Good	27 (71.1)	11 (28.9)	0.623
	Poor	36 (76.6)	11 (23.4)	

*Significant P value (P < 0.05). ^Φ^Fisher exact test was performed for this factor. MYR, Malaysian Ringgit; (USD 1 = MYR 4.15).

### Correlation between knowledge, attitude and practice

The correlation test found a significant positive correlation between knowledge-attitude (r_s_ = 0.384, P < 0.001), knowledge-practice (r_s_ = 0.319, P < 0.001) and attitude-practice (r_s_ = 0.457, P < 0.001). However, the degree of correlation was fair (r_s_ < 0.5). With further analysis, it was found that participants who had good knowledge were 2.8 times more likely have good attitude (OR:2.89; 95% CI:1.94–4.29) but no strong association was found with good practice (OR:1.96 ;95% CI:1.33–2.89) regarding dengue prevention. Nonetheless, participants with good attitude are 2.5 times more likely to have good dengue preventive practice.

## Discussion

This study provides the first description of KAP and seroprevalence in a closed, dengue endemic urban community in Malaysia, where an upward trend of dengue cases has been reported for more than a decade. Currently, Malaysia like many other countries in the region, are plagued by dengue. Dengue has no cure, only symptomatic management, while the current vaccine has moderate efficacy and does not provide equal protection against all four serotypes^1^. Hence, vector control remains the current mainstay for dengue control. More effort is needed on health campaign programs to educate the populace about prevention of dengue infection and to actively put these into action. Human practice is known to play a crucial role in maintaining dengue vector and transmission of virus because *Ae. aegypti*, the primary dengue vector depends on human to provide a suitable environment and for a blood meal. The latest KAP study in Malaysia by Ghani *et al*. (2019) reported that participants from non-dengue hotspot areas have better knowledge and attitude than those from hotspot areas but factors associated with this were unanswered^27^. The current study focuses more on the KAP level of residents from dengue hotspot and their dengue seroprevalence, along with factor associated with these variables. The findings from this study may contribute to the development of a proactive program to protect the health of vulnerable groups in the community.

It is noteworthy that interpretation and comparison of the data from this current study and from others have to be made cautiously. This is due to the methodological differences between studies including different modes of analyses of the data, varying focus of the questions in the questionnaire, dissimilar demographic background of the respondents, different scoring systems or cut-off points for “poor” and “good” KAP, etc. In view of the above, results from the current study were compared to those of previous studies on adults from urban/suburban settings similar to the current study. In our study, our cut-off values can be considered high i.e. 80% score, compared to studies which uses mean or other arbitrary cut-offs. Therefore, despite obtaining correct and positive answers (average of >75%) in each of the knowledge, attitude and practice component, only 50.7%, 46.8%, and 49.8% of the community living in the 8 apartments in Damansara Damai, a dengue hotspot, had good knowledge of dengue, attitude and practices in dengue prevention, respectively. Results of previous Malaysian studies showed that the urban/suburban communities generally have good knowledge of dengue and its symptoms and positive attitude on dengue prevention, but these are not translated to good practice in dengue prevention^25,30–32^. However, there are other studies which demonstrated various levels of KAP different from those reported above^26,28,33^, with few others citing good dengue prevention practices among the urban/suburban communities^24,34,35^.

This KAP study revealed that only half of the study population has exemplary and adequate knowledge regarding dengue. Nonetheless, it should be noted that the overall community did have exemplary knowledge of dengue infection per se and the signs and symptoms. This is consistent with earlier cross-sectional studies in Malaysia^25,36^, Jamaica^37^, Philippines^38^ and Thailand^39^. But it differs from some studies conducted in Nepal^40^ and India^41^. The difference may be due to intensified education and awareness campaign by the department of health in our endemic area which can be reflected in the communities’ level of knowledge. However, knowledge of the vector’s habit, behaviour and life-cycle is still lacking. This has been equally expressed in other similar studies^42,43^.

In addition, the current study found a lack of knowledge on transmission of dengue during pregnancy from mother to foetus among the study population. Hitherto, no KAP studies have assessed knowledge of vertical transmission of dengue, thus creating a dire void of knowledge on this issue. From a systematic review on maternal dengue and pregnancy outcome, most case reports or case series concluded that dengue infection before and even during pregnancy leads to adverse foetal outcomes due to vertical transmission of antibodies^44^. This is extremely worrying as seroprevalence analysis revealed 74.1% of 85 adults in the study population had previous exposure to dengue, without them knowing. Putting the two and two together, pregnant women may be unknowingly infected with dengue, but this vulnerable population and the foetus are at risk of complications during pregnancy, due to inapparent dengue. Indeed, a 2.5–3.4% rate of recent dengue infection (based on dengue IgM serology or RT-PCR) has been reported among pregnant women or parturient in Malaysia^15,45,46^ and New Caledonia^20^. Thus, it is important to heighten education among parents and future parents regarding the risks of dengue infection during pregnancy.

In univariate analysis, factor associated with good knowledge on dengue fever were being married, higher education level, being employed with higher monthly income and being Malay. However, the multivariate analysis revealed only race and employment were independent predictors of good knowledge. Many studies support a significant positive association between education and good knowledge of dengue^27,33,34,43^, while several studies support the effect of employment on knowledge^28,42^. This could be because working adults are more likely to be involved in health campaigns and education in their workplace and have more information on dengue fever compared to the unemployed. The Communications for Behavioural Changes (COMBI) programme on dengue prevention have also been implemented by Health Departments and developers at construction sites while the Department of Occupational Safety and Health of Malaysia do promote dengue awareness and prevention^47^. Obviously, there is a direct relationship between economic statuses (e.g. owning a house, better income) and having good knowledge of dengue^28,33,35,48^. People with better economic status may have better access and appreciation for reliable information. Other studies have also described significant associations between good knowledge scores of dengue with being married^28,42^, increasing age^43^ and history of dengue infection^33,34^.

Poor attitude (53.2%) and poor practice (50.2%) were observed among participants in the study area. Many (49.6%) have an erroneous belief that chemical fogging by the health personnel is adequate to reduce dengue transmission, similar to results reported by Kamel *et al*. (2017). They do not realise it is important to carry out source reduction since in Malaysia, fogging is always carried out after cases are reported. Chemical fogging itself has many pros and cons as a control measure^49,50^. From the ongoing cluster randomised trial using GOS trap and NS1 antigen test kit (unpublished data, 2020), we were still able to detect mosquitoes with dengue virus after fogging was carried out. This concept must change, and more proactive measures are needed. Vector control cannot rely on fogging alone, effective vector control requires reduction of vector breeding habitats to reduce disease transmission and prevalence. Moreover, evidence has been provided that ULV is not effective^51^, yet it seems to be the main strategy used during outbreaks. Perhaps, that is the reason why residents think that it is the best method for getting rid of the mosquitoes.

Furthermore, there was a small proportion of participants who believed that it is not their responsibility to remove mosquito breeding sites in their residence. This is similar to that of Zaki *et al*. (2019) and of another study which states that 32.7% believed removing larvae breeding is a complete waste of time^23^. In order to curb dengue, reducing the vector population and prevention of virus transmission are equally important. Without community participation, it is impossible to reduce dengue prevalence. In multi-storey apartments, all residents should a play role to clean up their housing units to ensure it is free of mosquito breeding sites. As people are living very close to each other, they can be bitten by infected mosquitoes which can easily fly from one house to another. Also, since most of the dengue epidemics are occurring in high-rise apartments, the management bodies of the apartments should take the responsibility of keeping the surroundings free of breeding sites while the residents should take care of their homes. Previous study has reported that search and destroy practice requires good knowledge and skills in order to remove breeding sites efficiently^52^. Therefore, the health authorises have a role in spearheading this effort too. Nonetheless, it is encouraging to note that the general public, including those in the current study support dengue control programs and believe that the public also has the responsibility in preventing and controlling dengue^10,25,26,36,43^.

In univariate analysis, factors associated with good attitudes were age, race, marital status, employment status and monthly individual income. These factors can be categorised as good socioeconomic background. The findings support the notion that participants with better socioeconomic background have good attitude regarding dengue fever. Other Malaysian studies have reported income^27,31,35^, employment status^27^, marital status^53^ and ethnicity (especially being Malay)^43,54^ to be associated with good attitude of dengue prevention. Similarly, in Aceh, different areas have reported that good attitude was associated with socioeconomic status. Better socioeconomic status does provide better access to dengue information^55,56^. However, excluding all insignificant variables, race, marital status and employment are independent predictor of good attitudes. Married couples possess good attitudes towards dengue control than single individuals. This may be due to greater sense of responsibility towards their family compared to single inhabitants who may be staying there temporarily for working purposes. A study in Lao PDR described that families tend to possess more resources to make sure their house and surroundings are comfortable, safe for the children and clear of *Aedes* breeding sites^57^. Conversely, findings from Singapore have further shown that a high incidence of dengue fever was associated with living alone and having no family nucleus^58^.

Interestingly, in multivariate analysis, race was a strong significant predicator for good knowledge, attitudes and practices. The Malay race possessed good KAP in comparison to other races. Two studies have shown Malays outperformed other ethnic groups in this context^30,54^. These may be due to the main language used in most mass media. Mass media is a powerful tool in disseminating health information and consistent with previous studies done in Malaysia^23,44^, Indonesia^56^ and Thailand^59^. In Malaysia, most mass media advertisements about health awareness are mainly in the national language understood by the Malay race more so than the other races. Other studies have also indicated the Malay race to be associated with good practice of dengue prevention^43,54^. Nonetheless, there might be underlying actual characteristics behind the variable race for being associated with good KAP. For example, being pious to religion or having a strong sense of community. Perhaps there is a better, ingrained sense of community attributed to the more religious and cultural upbringing of the Malays than among the other races. Interestingly, this phenomenon is most apparent in rural settings, where the villagers are mostly Malays with a strong sense of neighbourliness, and they often demonstrate good dengue prevention practices^36,60,61^.

From seroprevalence results, it was noted that dengue IgG seropositivity was significantly associated with race and type of buildings. Additionally, 75.0% of the participants with positive dengue IgG possessed poor knowledge, attitude and practice toward dengue disease and prevention. 83.0% of residents from shop houses were dengue IgG seropositive in this study area. This can be explained by population density and mobilization effects, as shop houses are typically surrounded by shops where high population movement occurs and thus residents are at higher risk of imported dengue. In fact, mosquitoes tend to remain in the same location throughout their lifetime. This means that people, rather than mosquitoes are responsible for dengue transmission in and outside of their communities^62^. Dense population has been postulated as one of the factors causing transmission of dengue virus^29,63^. Hence, a significant positive association between type of residential unit with dengue IgG seropositivity found in this study could be linked to the interplay of abundant mosquito breeding sites^64,65^, high population density and human mobility, leading to high dengue incidence attributed to import dengue. Wong *et al*. (2014) showed that significantly higher proportion of people living in high-rise residential buildings are IgG seropositive, compared to people living in single or terraced houses and they experience more frequent fogging and mosquito problems^29^.

These self-reported questionnaires do show a fair correlation between means score of knowledge, attitude and practice. As knowledge improves, attitude and practice among participants also improve in the study area. Moreover, a significant positive association was observed between knowledge, attitude and practice. Our results are most similar to some earlier studies^26,43^. Whereas other studies only reported correlation between knowledge on dengue with positive attitude for dengue control^53^ and between positive attitude and good practice of dengue prevention^33,35^. However, the level of translation from knowledge to attitude and practice of the community in this study is still low. Even with good knowledge, poor attitude and practice were observed in the study population in some instances. Evidence of association between knowledge with practice is varying. While some observed a positive association in Malaysia^29,31,42^, Cuba^48^ and Laos^66^,others saw no correlation between good knowledge and practices^28,32,67^. In our current study, an effective and sustainable strategy is required to translate the community’s knowledge into good practices.

Overall, to tackle, poor attitude and practice in our study population, change in their behaviour towards dengue prevention is very crucial. The residents have fairly good knowledge, but practice and attitude need improvement. Perhaps, the residents have always been used to reactive methods since they were only informed when dengue cases occurred. And actions are taken only when being monitored or when there is a death case. Also, possibly some people do perform good practices but quickly became demotivated when they realise their efforts are not matched by their immediate community^68^. Therefore, heightening awareness and good attitude and positive encouragement of subsequent good practices are important. Seeing results (eg reduction in *Aedes* population or dengue cases) of their preventive behaviours may fuel continuous motivation to perform these activities^69^. Besides, it is envisaged that with the new proactive paradigms like informing residents when dengue positive mosquitoes are obtained may improve their attitude and practice and to carry out source reduction^70^.

Knowledge and attitude are associated with practice, and these two are easier to improve than to improve other factors associated with good practice such as economic status^71^. Besides, ingrained, negative habits are difficult to discourage with plain knowledge sharing. Perhaps more personal and practical approaches in the health education programmes are needed to influence a change in behaviour. This has been a common recommendation by previous studies since the early 1990’s^34,64,70^. House-to-house inspection by health personnel should not be done for *Aedes* surveillance only, but to convey information and educate the residents in a more personal manner. Health personnel and religious bodies should be encouraged to influence and motivate change in habit and spur social mobilization^10,23,72^. Furthermore, the provision of adequate and relevant information should be made readily available to all layers of the communities. For this matter, television/radio has been cited as the main source of information, followed by newspaper/magazine in both the urban and rural communities^30–32,43^.

Finally, some limitations of this study is that the subjects were recruited with more than one approaches such as from house to house and community event like clean-ups causing non-probabilistic sampling. Their KAP levels are also assessed only at one time point, so the overall dynamic might change according to time. Moreover, as a self-reporting questionnaire was used, respondents might had provided answers not reflective of their actual attitude and practices, to appear socially desirable, which may contribute to reporting bias.

Therefore, this study revealed knowledge regarding dengue symptoms, transmission (except vertical transmission and dengue during pregnancy) and vector control measures to be generally high among the residents of the 8 apartments in the dengue hotspot, Damansara Damai, Petaling Jaya, Selangor, Malaysia. Furthermore, dengue IgG seropositivity was significantly associated with race and living in shop houses. However, this knowledge on dengue and prevention is not translated to positive attitude and practice. In this sort of setting, conventional health education campaigns should be adapted to encourage social mobilization among the people. Bottom-up approaches are more likely to be successful and sustainable^73^. What is essential is a multi-disciplinary approach to change the attitudes and behaviour of the people. This can be achieved by having many different stakeholders coming together, synergising their efforts to fight a common cause. To combat dengue successfully without greater harm to the ecosystem, integrated vector control measures will rely mostly on the success of community and household practices to eliminate vector breeding sites.

## Methodology

### Study setting

**Figure 1:**
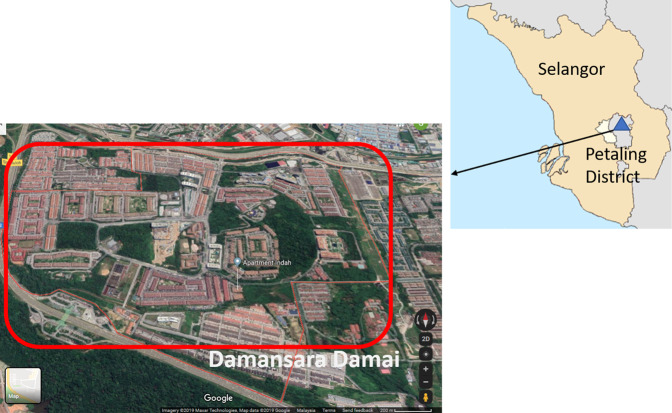
Map of Selangor state, Malaysia showing study district, Petaling district. Damansara Damai is one of the sub-district which is a closed area with only one main entrance and exit.

A community-based, cross-sectional study was conducted at Damansara Damai (3.1930°N, 101.5923°E), a residential area located at the northernmost part of Petaling Jaya, the district, which accounted for the largest number of dengue cases in the state of Selangor^6^. Damansara Damai (Fig. 1) has a population of 61,615 in an area of 3.45 km^2^ which translates into a population density of approximately 17,859 inhabitants/km^2^. Overpopulation in the low and high-rise residential buildings may be responsible for the major dengue hotspots in the study site. A detailed description of the study site has been published^70^. This cross-sectional study was conducted during a cluster randomized controlled trial study investigating the efficacies of Gravid Ovipositing Sticky Trap (GOS) and dengue NS1 antigen test kit as early surveillance tool for dengue. This trial has been registered at ClinicalTrials.gov (ID: NCT03799237) on 8th January 2019. A detailed study protocol is available online^70^.

### Ethic approval

This research received the ethical approval from Medical Research Ethics Committee, University of Malaya Medical Centre, Malaysia (MRECID No: 2018525-6321).

### Participation and sample size

Sample size estimation was calculated to achieve power of 80% with a 5% margin of error and 95% confidence level, by using the Cochran (1963) formulae^72^. Since the prevalence of KAP in the study population is unknown, the estimated sample size was calculated by assuming that 50% of the population have baseline knowledge about dengue. A minimum of 384 residents were required for this study; a further 20% was added to account for anticipated loss of participants and this increased the sample size to 460 residents. Data was collected from September 2018 to January 2019. The residents were approached during office hours on weekdays by a maximum of two research teams, each of which has at least a well-trained phlebotomist. Specifically, house-to-house visits were performed following receipt of written approval by the joint management bodies of respective apartments. All residents who were aged at least 18 years and could provide informed consent were included in the study. The research followed the guidelines stated in the ethics form and written informed consent was obtained from all participants.

### Knowledge, attitude, and practice questionnaire

A self-administered close-ended questionnaire was used as the instrument to measure the residents’ baseline KAP. The questionnaire has been adapted from previous study^40^ and used in a pilot study which involved similar Malaysian community^25^. There are three sections in the questionnaire. Section A concerns the sociodemographic data which covers the basic information of respondents. Section B contains 28 questions to assess the residents’ knowledge on dengue. The knowledge possessed by a community refers to their understanding towards dengue including its vectors and symptoms of dengue. Section C has 9 attitude-related items referring to their feelings as well as any preconceived idea of dengue, and 7 items on common dengue prevention practices of the respondents. Practices refer to the ways in which they demonstrate their knowledge and attitude through their actions.

### Dengue seroprevalence test

In addition to the KAP survey, the respondents were also given the choice to participate in the seroprevalence study. Respondents name, identification number and consent were taken before 3 ml of venous blood was withdrawn by the trained personnel. The samples were collected in EDTA blood tubes and transported to the laboratory at room temperature within 4 hours. The blood was then centrifuged at 4,000 rpm for 4 min and the resulting serum aliquoted, coded and stored at −80 °C pending further use. Later, Dengue IgM and IgG ELISA kit (Focus Diagnostics Inc., Cypress, CA, USA) were used to detect the presence of anti-dengue immunoglobulin in the sera according to manufacturer’s instructions. Each individual’s serum was tested in duplicates.

### Data management and analysis

Each resident’s KAP questionnaire and sample were coded with a unique identification number based on their apartments. Data analysis was performed using Statistical Package for Social Science version 23 (SPSS, Inc., Chicago, IL). A scoring system was utilized in the evaluation of the KAP data. Specifically, all correct and positive answer was scored 1 while “Don’t Know” and wrong answers as 0. The total number of correct answers in each section was used to determine the KAP level. If a participant scored at least 80% in a category, he/she would be labelled as “good”; if the converse was true, then he/ she would be labelled as “poor”. Scoring system and cut-off points for the KAP survey were in accordance with those of Dhimal *et al*. (2014)^40^.

Descriptive statistics of the sociodemographic factors comprised frequencies and percentages. The association of independent variable with KAP levels (good/poor) were determined using the chi-square test or Fisher’s exact test as appropriate. Meanwhile, Spearmen’s rank correlation was used to determine the extent of correlation between KAP scores since data was not normally distributed as per outcome of the Kolmogorov-Smirnov normality test. The level of statistical significance was set at 0.05. Univariate analysis was performed to examine the association between good KAP and the demographic and socioeconomic factors. Then, multivariable logistic regression model was developed with all possible associations, variable that showed an association with P ≤ 0.25 were included in the model, as suggested by Bendel and Afifi^74^.

## Data Availability

The analysed data is all in the manuscript. However, the dataset of this study is available upon request from the corresponding author.
